# Possible Contribution of Taurine to Distorted Glucagon Secretion in Intra-Islet Insulin Deficiency: A Metabolome Analysis Using a Novel α-Cell Model of Insulin-Deficient Diabetes

**DOI:** 10.1371/journal.pone.0113254

**Published:** 2014-11-13

**Authors:** Megumi Bessho, Yuko Murase-Mishiba, Akihisa Imagawa, Jungo Terasaki, Toshiaki Hanafusa

**Affiliations:** 1 Department of Internal Medicine (I), Osaka Medical College, Osaka, Japan; 2 Department of Metabolic Medicine, Graduate School of Medicine, Osaka University, Osaka, Japan; University of Lille Nord de France, France

## Abstract

Glycemic instability is a serious problem in patients with insulin-deficient diabetes, and it may be due in part to abnormal endogenous glucagon secretion. However, the intracellular metabolic mechanism(s) involved in the aberrant glucagon response under the condition of insulin deficiency has not yet been elucidated. To investigate the metabolic traits that underlie the distortion of glucagon secretion under insulin deficient conditions, we generated an αTC1-6 cell line with stable knockdown of the insulin receptor (IRKD), i.e., an *in vitro* α-cell model for insulin-deficient diabetes, which exhibits an abnormal glucagon response to glucose. A comprehensive metabolomic analysis of the IRKD αTC1-6 cells (IRKD cells) revealed some candidate metabolites whose levels differed markedly compared to those in control αTC1-6 cells, but also which could affect the glucagon release in IRKD cells. Of these candidates, taurine was remarkably increased in the IRKD cells and was identified as a stimulator of glucagon in αTC1-6 cells. Taurine also paradoxically exaggerated the glucagon secretion at a high glucose concentration in IRKD cells and islets with IRKD. These results indicate that the metabolic alterations induced by IRKD in α-cells, especially the increase of taurine, may lead to the distorted glucagon response in IRKD cells, suggesting the importance of taurine in the paradoxical glucagon response and the resultant glucose instability in insulin-deficient diabetes.

## Introduction

Glycemic instability is a crucial clinical problem in patients with insulin-deficient (type 1 and advanced type 2) diabetes. The wide fluctuations of glucose are due not only to the insulin deficiency, which has been considered to be one of the leading causes of glycemic volatility [1], [2], but also may be at least partly due to abnormal glucagon secretion [3]; namely, a deficient glucagon response to hypoglycemia [4] and an inappropriately high glucagon response to hyperglycemia [5]. However, the effects of distorted glucagon secretion on the glycemic excursion have been largely overlooked. We recently reported that arginine-stimulated glucagon secretion is positively associated with the degree of glucose fluctuation in type 1 diabetic patients whose endogenous insulin was completely depleted [6]. Therefore, the aberrant increase in glucagon might contribute to glycemic instability, independent of deficient endogenous insulin.

However, little is known about the pathogenesis of the aberrant glucagon response in the pancreatic α-cells in insulin-deficient type 1 diabetic patients, which has so far been attributed to altered neuronal control [7], impairment of intrinsic glucose sensing by the α-cells themselves [8], and/or the local paracrine defects. Substances released from the neighboring endocrine cells, such as insulin, islet amyloid polypeptide, Zn^2+^, ATP and GABA from β-cells, and somatostatin from δ-cells, have been reported to regulate glucagon secretion [9]–[12]. Of all these molecules, the actions of insulin or its signaling pathway may be key modulators for α-cell function, because extensive physiological and molecular biological approaches have demonstrated its importance among the mechanisms that regulate glucagon secretion in an intra-islet manner [13]. Indeed, hyperglucagonemia is suggested to develop in parallel with hypoinsulinemia [14]. The suppression of insulin signaling by insulin receptor (IR) silencing through a siRNA approach in α-cells has been reported to disturb the glucagon secretion in response to glucose [15]. In addition, an α-cell-specific insulin receptor knockout mouse has been demonstrated to exhibit an exaggerated glucagon response under both normo- and hypoglycemic conditions [16]. These studies strongly suggested that a lack of paracrine control by insulin on α-cells could account for the dysregulated glucagon secretion in insulin-deficient type 1 diabetes, although the intracellular metabolic mechanism(s) involved have not been elucidated.

To explore the cellular metabolic changes in α-cells under pathophysiological conditions of insulin-deficient diabetes, we generated a clonal mouse αTC1-6 cell line with a stably knocked-down IR, as a model of α cells in insulin deficiency, and performed a comprehensive *in vitro* intracellular metabolomic analysis. We herein provide evidence that metabolic alterations in the α-cell model of insulinopenic diabetes may lead to a paradoxical glucagon response, which would thereby lead to glycemic instability in insulin-deficient type 1 diabetes.

## Materials and Methods

### Cell culture

αTC1-6 cells (kindly provided as a gift by Dr. Y. Moriyama, Okayama University, Japan) [17]–[19], a mouse α-cell line, were used in the present study. The αTC1-6 cells were originally isolated from αTCl cells, an α-cell-derived multiclonal cell line established using a transgene strategy [17]. Although the original αTCl cells were composed of heterologous cell populations that seemed to contain α-cells and their progenitors, the αTCl-6 clone totally lacked insulin mRNA, and thus was similar to the differentiated α-cells *in vivo* [17]. The cells were maintained in DMEM (Invitrogen Inc., Carlsbad, CA) containing 25 mM glucose supplemented with 10% FCS (Sigma-Aldrich, St. Louis, MO), 100 units/ml penicillin, 100 µg/ml streptomycin, 0.25 mg/l fungizone (Invitrogen) and 55 µg/ml sodium pyruvate at 37°C in a humidified atmosphere (5% CO_2_, 95% air).

### Islet preparation and culture

Islets from male ICR mice (aged 7–8 weeks) were obtained from Cosmo Bio Co., Tokyo, Japan. Briefly, islets were isolated by the collagenase digestion of the pancreas as described previously [20], with some modifications. The animals were anesthetized by the intraperitoneal injection of pentobarbital sodium at 80 mg/kg. The abdomen was opened, and collagenase (3 mg/ml) dissolved in 6 ml of 5 mM Ca^2+^-containing Krebs-Ringer bicarbonate buffer solution was injected into the common bile duct at the distal end after ligation of the duct proximal to the pancreas. The pancreas was dissected out and incubated at 37°C for 17 min. Isolated islets were handpicked and cultured in RPMI-1640 media (11 mM glucose) at 37°C and 5% CO_2_. This study was conducted under the approval of the institutional animal care and use committee of Osaka Medical College.

### Lentiviral transduction to generate shRNA-mediated stable knockdown cell lines and primary islets

MISSION lentiviral transduction particles expressing short hairpin RNA (shRNA) targeting the IR (TRCN0000023574, TRCN0000023575, TRCN0000023576, TRCN0000023577, TRCN0000023578) or a scrambled non-target negative control (SHC002V) were purchased from Sigma-Aldrich. Stably transduced cell lines were generated according to the manufacturer's instructions. Briefly, cells were seeded on 96-well plates. On the following day, the cells were infected with 50 MOI (multiplicity of infection) of the lentivirus. After 24 h, the medium was changed. The selection of stable clones started 24 h later with the addition of 1 µg/ml of puromycin. Surviving clones were screened for mRNA knockdown. Of the five IR-targeted lentiviral clones tested, the maximal knockdown was achieved with clone TRCN0000023575 (CCGGCCCTGAAGGATGGAGTCTTTACTCGAGTAAAGACTCCATCCTTCAGGGTTTTT). This clone was therefore selected and used for further studies. We performed functional assays 72–96 h post-transduction to determine the optimal transduction efficiency using the MISSION TurboGFP Control Transduction Particles (SIGMA) at various MOI (1, 5, 10 and 50) before assaying them with shRNA constructs. As a result, a MOI of 50 was found to be the best for transduction, with transfection efficiencies that reached >80%. We did not observe any non-specific cell death at a MOI of 50.

For islets, lentivirus infection was carried out the day after isolation. Briefly, isolated islets were plated in 96-well plates, 10–20 islets/well, with 120 µl of RPMI islet culture medium containing 11 mM glucose for 1–2 h at 37°C. The islets were infected with 20 MOI of the lentivirus expressing shRNA targeting IR (TRCN0000023575) or the scrambled sequence control shRNA (SHC002V), then were incubated overnight at 37°C. On the following day, the islets were transferred to new plates, washed twice and further cultured at 37°C, with the medium changed on a daily basis. Functional assays were performed 72 h post-transduction. The transduction and transfection efficiencies, as determined by GFP fluorescence, reached >80% under these conditions.

### Western blot analysis

For extraction of the total protein, cell samples were homogenized in ice-cold lysis buffer (Cell Signaling Technology, Beverly, MA). Lysates were subjected to SDS-PAGE and blotted using antibodies against insulin receptor-β (Santa Cruz Biotechnology, Santa Cruz, CA), β-actin, Akt, phospho-Akt (Ser473), mammalian target of rapamycin (mTOR), phospho-mTOR (Ser2448), AMP-activated protein kinase (AMPK) α, phospho-AMPKα (Thr172), glycogen synthase kinase-3β (GSK-3β), phosho-GSK-3β (Ser9), extracellular signal-related kinase 1/2 (Erk1/2) or phospho-Erk1/2 (Thr202/Tyr204) (Cell signaling Technology). The densitometric analysis was performed using the Image J software program.

### Insulin stimulation

Insulin receptor knock down (IRKD) and scrambled non-target negative control (control) αTC1-6 cells were plated in 10 cm culture plates at 80% confluency, and were serum starved for 24 h in DMEM. Subsequently, the cells were stimulated with 100 nM insulin for 15 min at 37°C in a humidified atmosphere (5% CO_2_, 95% air). After the reaction was stopped by washing the cells twice with ice-cold PBS, we added 500 µl ice-cold lysis buffer (Cell Signaling Technology) and collected samples from each dish in microtubes. Cell lysates were prepared as described above for the Western blot analysis.

### Cell viability, cytotoxicity and apoptosis assays

The cell viability (GF-AFC cleavage), cytotoxicity (bis-AAF-R110 cleavage) and apoptosis (caspase 3/7 activity) were measured simultaneously via the Apotox-Glo Triplex assay (Promega, Madison, WI). IRKD cells and control cells were plated (10^4^ cells/100 µl/well) in 96 well plates in complete growth media. Samples were assayed 0, 1, 2, 4 and 6 days after plating, as indicated, and were processed according to the manufacturer's instructions. The GF-AFC and bis-AAF-R110 substrates were added, and fluorescence was measured by the GloMax-Multi Detection System (Promega) (excitation 400 nm/emission 485 nm and 505 nm/520 nm, respectively) after a 30 min incubation at 37°C. Luminescence was detected after a 30 min incubation with the Caspase-Glo 3/7 Substrate (Promega). After the background for each assay was subtracted, the data were represented as the fold-change from baseline (day 0).

### Cell proliferation assay

Cell proliferation was measured by the BrdU (5-bromo-2′-deoxyuridine) incorporation assay using a CycLex Cellular BrdU ELISA Kit (CycLex, Nagano, Japan) according to the manufacturer's instructions. Briefly, IRKD and control cells (10^4^ cells/100 µl/well) were cultured in 96-well plates in complete growth media. Samples were assayed 1, 2, 3 and 4 days after plating, as indicated. BrdU, at a concentration of 10 µM, was added to the medium and incubated for another two hours. Denatured cells were exposed to an anti-BrdU monoclonal antibody, followed by horseradish peroxidase-conjugated goat anti-mouse IgG. The reaction product was quantified by measuring the absorbance using a scanning multi-well spectrophotometer (ELISA reader) at 450 nm with a reference wavelength of 540 nm.

### Glucagon secretion assay

Both IRKD αTC1-6 cells (IRKD cells) and control αTC1-6 cells were plated in 24-well culture plates at 80% confluency. Forty-eight hours later, the cells were preincubated in Krebs-Ringer bicarbonate buffer (KRB) containing 115 mM NaCl, 5 mM KCl, 24 mM NaHCO_3_, 2.5 mM CaCl_2_, 1 mM MgCl_2_, 10 mM HEPES and 2% bovine serum albumin with 5.6 mM glucose for 1 h at 37°C. Subsequently, the cells were treated with 1000 µl of KRB buffer containing either 1.5 mM, 5.6 mM or 30 mM glucose for 2 h at 37°C. Aliquots (500 µl) from each treatment were taken at the end of the 2-h incubation to assess the glucagon secretion by an ELISA (YK090 Glucagon EIA; Yanaihara Institute Inc., Shizuoka, Japan). Following the secretion assay, the cellular glucagon content was assessed by acid ethanol extraction (0.15 M HCl in 75% ethanol in H_2_O). The secretion data and content data from the assays were normalized to the total protein content measured by the bicinchoninic acid (BCA) protein assay (Pierce BCA Protein Assay Kit; Thermo scientific, Rockford, IL). For the taurine and/or insulin supplementation experiments, the cells were prepared as described above, and then were subsequently supplemented with taurine and/or insulin for 2 h. The taurine treatment for the control αTC1-6 cells was up to100 mM based on the fact that the plasma taurine concentrations in most vertebrate species are usually lower than 1 mM, while the intracellular taurine concentration ranges from 10 to 50 mM [21], and the concentration of added taurine was in the tens of millimolar range in some previous *in vitro* experiments [22].

Batches of 10–20 isolated islets with IRKD or transfected with the control were preincubated for 60 min at 37°C in a humidified atmosphere containing 5% CO_2_ in 500 µl KRB buffer supplemented with 5.6 mM glucose. The islets were then incubated for 2 h at 37°C with 500 µl of KRB buffer containing 1.5 mM, 5.6 mM or 30 mM glucose. Finally, the glucagon secretion and intracellular contents were assessed by the above-mentioned ELISA, and these values were normalized to the islet numbers.

### Metabolome analysis

IRKD and control αTC1-6 cells were plated in 10 cm culture plates at 80% confluency. Forty-eight hours later, the cells were preincubated in KRB buffer with 5.6 mM glucose for 1 h at 37°C. Subsequently, the cells were treated with KRB buffer containing 1.5 mM, 5.6 mM or 30 mM glucose for 2 h at 37°C. The culture medium was aspirated from the dish, and the cells were washed twice with a 5% mannitol solution (10 ml first and then 2 ml). The cells were then treated with 800 µl of methanol and left to rest for 30 sec in order to inactivate enzymes. Next, the cell extract was treated with 550 µl of Milli-Q water containing 10 µM internal standards (H3304-1002, Human Metabolome Technologies, Inc., Tsuruoka, Japan) and left to rest for another 30 sec. The extract was obtained and centrifuged at 2,300×g and 4°C for 5 min, and then 800 µl of the upper aqueous layer was centrifugally filtered through a Millipore 5-kDa cutoff filter at 9,100×g and 4°C for 120 min to remove proteins. The filtrate was centrifugally concentrated and re-suspended in 50 µl of Milli-Q water for a CE-MS analysis. Metabolome measurements were carried out by Human Metabolome Technology Inc., Tsuruoka, Japan.

The CE-TOF-MS was carried out using an Agilent CE Capillary Electrophoresis System equipped with an Agilent 6210 time-of-flight mass spectrometer, an Agilent 1100 isocratic HPLC pump, an Agilent G1603A CE-MS adapter kit and an Agilent G1607A CE-ESI-MS sprayer kit (Agilent Technologies, Waldbronn, Germany). The systems were controlled by the Agilent G2201AA ChemStation software program, version B.03.01 for CE (Agilent Technologies, Waldbronn, Germany). The metabolites were analyzed by using a fused silica capillary (50 µm i.d.×80 cm total length), with commercial electrophoresis buffer (Solution ID: H3301-1001 for the cation analysis and H3302-1021 for the anion analysis, Human Metabolome Technologies) as the electrolyte. The sample was injected at a pressure of 50 mbar for 10 sec (approximately 10 nl) in the cation analysis and 25 sec (approximately 25 nl) in the anion analysis. The applied voltage was set at 27 kV. ESI-MS proceeded in the positive ion mode, and the capillary was set at 4,000 V. The spectrometer was scanned from a mass/electric charge ratio (*m/z*) of 50 to 1,000. The other conditions used were the same as were described previously [23]–[25].

The raw data obtained by CE-TOF-MS were processed using the MasterHands ver. 2.13.0.8.h software program (developed at Keio University, Japans). Signal peaks corresponding to isotopomers, adduct ions and other product ions of known metabolites were excluded, all signal peaks potentially corresponding to authentic compounds were extracted and then their migration times (MT) were normalized against those of the internal standards. Thereafter, peaks were aligned according to the *m/z* and normalized MT values. Finally, the peak areas were normalized to those of the internal standards, methionine sulfone and D-Camphor-10-sulfonic acid, for cations and anions, respectively. The resultant relative area values were further normalized by the cell count. All experiments were conducted independently three times.

### Quantitative real-time PCR

Total RNA isolation and cDNA synthesis were performed using the TaqMan Gene Expression Cell-to-CT Kit (Applied Biosystems, Foster City, CA) according to the manufacturer's protocol. The resulting pre-amplified cDNA preparations were analyzed by real-time PCR in a Step One Plus Real-time PCR System (Applied Biosystems) using the TaqMan Gene Expression Assay in combination with TaqMan Gene Expression Master Mix containing ROX (Applied Biosystems), according to the manufacturer's instructions. All of the probes were purchased from Applied Biosystems. The data were normalized to the β-actin level.

### Taurine and amino acid uptake assay

The taurine and amino acid uptake were measured by a previously described method, with the following modifications [26]: In brief, confluent cells in 24-well plates were preincubated in KRB buffer with 5.6 mM glucose for 2 h at 37°C. The cells were supplemented and incubated with tritiated taurine or amino acids (taurine, L-arginine, L-glutamine or L-leucine; 0.44 µCi/well) (American Radiolabeled Chemicals, Inc., St. Louis, MO), together with 1 mM unlabelled taurine or amino acids, for 1, 10, 30, 60, 90 or 120 min. The incubation was stopped by removing the incubation medium and washing the cells three times with ice-cold PBS. The cells were then lysed with 300 µl of 0.1% Triton X-100. The radioactivity in the whole-cell lysates was counted with a Packard Tri-Carb 2200 CA liquid scintillation analyzer.

### Statistical analysis

All data are the means ± SEM. Student's *t*-test or Welch's *t*- test was performed for comparisons of two groups, as appropriate. Comparisons of more than two groups were made using a one-way ANOVA. Differences were considered to be statistically significant at *P*<0.05.

## Results

### Generation of stable IR knockdown αTC1-6 cells and IR knockdown in mouse islets

**Figure 1:**
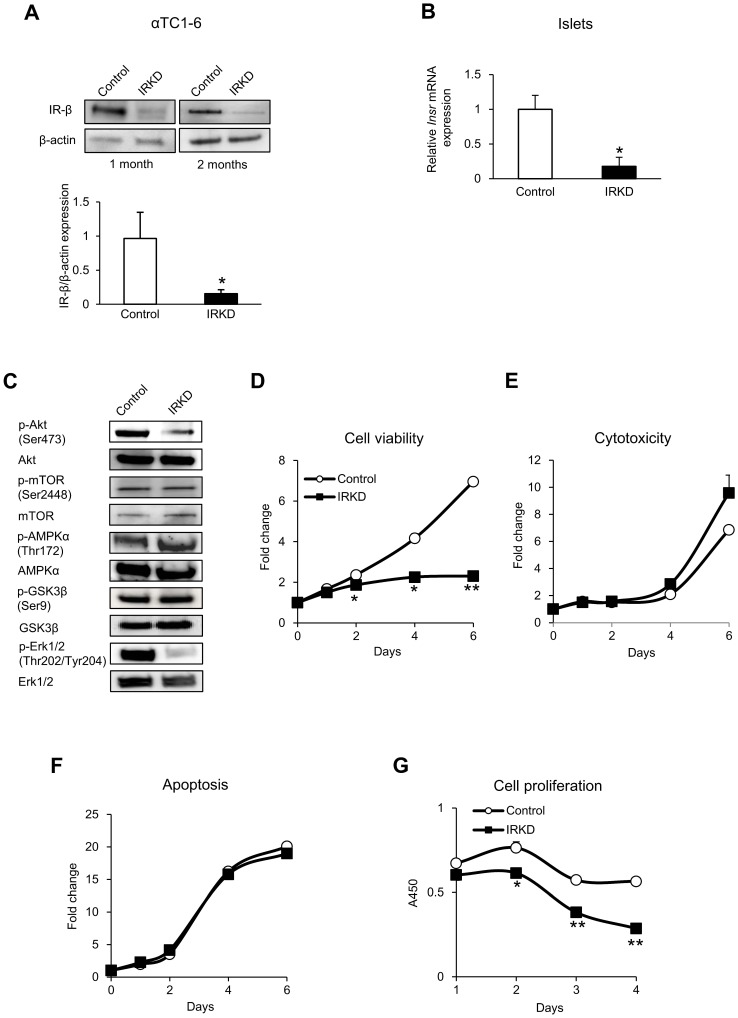
Results of an analysis of the effect of stable IR knockdown in αTC1-6 cells and IR knockdown in mouse islets. (**A**) An immunoblot analysis of the insulin receptor (IR) expression in αTC1-6 cells after the transduction with lentiviruses expressing control shRNA (control) and shRNA-IR (IRKD). αTC1-6 cells were infected with lentiviruses (see details in the “Materials and Methods”), and were cultured in medium containing 1 µg/ml puromycin for one month to generate homogenous stable cell lines. Cells were then analyzed by a Western blot analysis using specific polyclonal antibodies against the β-subunit of the IR (IR-β). Stable αTC1-6 cells maintained in culture for two months were also examined. Blots are shown on the *top*, and the relative amount of IRβ protein normalized to the β-actin level is shown on the *bottom* (*n* = 3. The bars represent the means± SEM; *P<0.05, control vs. IRKD). (**B**) The relative expression of the *Insr* gene in isolated mouse islets infected with lentiviruses expressing control and IRKD (*n* = 6. The bars represent the means± SEM; *P<0.05, control vs. IRKD). (**C**) The results of the immunoblot analysis of the activation of insulin signal transduction pathways in control and IRKD αTC1-6 cells. Cells were serum-starved for 24 h in DMEM and were subsequently stimulated with 100 nM of insulin for 15 min. Samples were analyzed by a Western blot analysis using specific antibodies against phospho- and non-phospho-proteins, as indicated. (**D–F**) The time course of the changes in cell viability, cytotoxicity and apoptosis assessed using the ApoTox-Glo Triplex Assay (Promega) in the control and IRKD cells. *n* = 3 in each group. The data are expressed as the means ± SEM; *P<0.05, **P<0.01, control vs. IRKD. (**G**) The time course of BrdU incorporation in the control and IRKD cells. *n* = 3 in each group. The data are expressed as the means ± SEM; *P<0.05, **P<0.01, control vs. IRKD.

To achieve stable expression of shRNA in cultured cell lines, a lentiviral vector was used to transduce the αTC1-6 cells. The effectiveness of knockdown in the stable cell lines was confirmed by a Western blot analysis using cells at six and 12 passages post-infection (one month and two months), and a maximum of 80% IR-knockdown at the protein level was observed in the cells at both of these passages (Fig. 1A). Similarly, isolated islets infected with a shRNA lentivirus targeting the IR showed an 82.1% decrease in *Insr* expression (Fig. 1B).

To evaluate the effect of the IR knockdown on insulin signal transduction, we examined the activation of pathways downstream of insulin signaling. The degree of insulin-induced phosphorylation of Akt was decreased in IRKD cells compared to control αTC1-6 cells, whereas the levels of mTOR, GSK3β and AMPKα were similar in αTC1-6 cells with and without IR (Fig. 1C). Erk1/2, a key mediator of the mitogenic potential of growth factors, was less activated in IRKD cells than in control cells, which was supported by the observation of reduced cellular proliferation in IRKD cells.

### Effects of IR knockdown on the viability, apoptosis, cytotoxicity and proliferation of αTC1-6 cells

The cell viability was significantly more decreased with time from day 2 in the IRKD cells compared to the control cells (Fig. 1D), although the levels of cytotoxicity and apoptosis were not significantly different between these two cell types (Figs. 1E and F). Cell proliferation was studied using the BrdU incorporation assay, and a significant reduction in the proliferation in IRKD cells was found (Fig. 1G). The observation that IRKD cells were significantly delayed in their transition from the G0/G1 to S phase, which was not accompanied by an increase in apoptosis, suggests that insulin signaling is responsible for cell cycle regulation in this cell line.

### Effects of IR knockdown on the glucose-regulated glucagon secretion in αTC1-6 cells and islets

**Figure 2:**
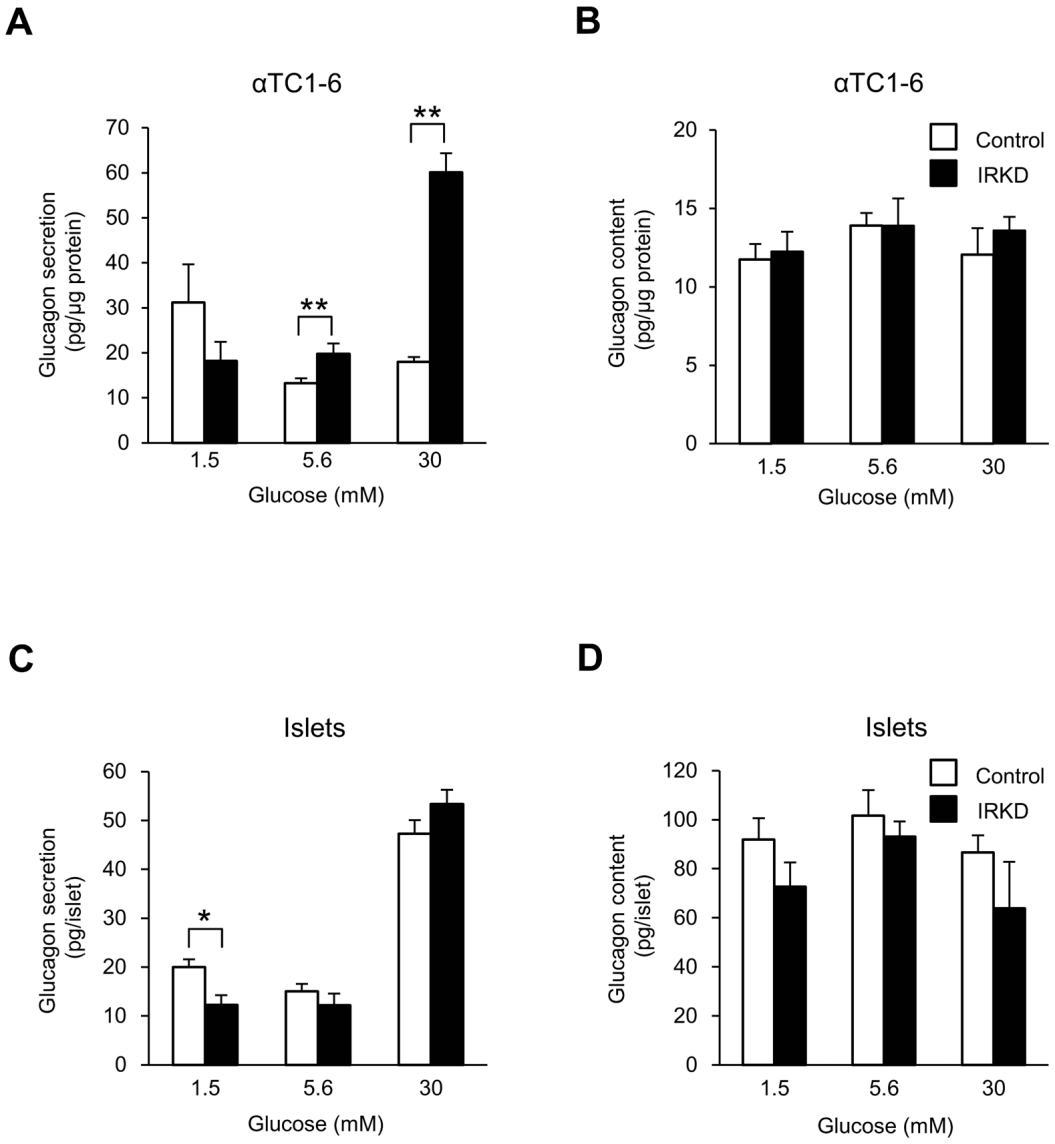
Glucose-stimulated glucagon secretion and cellular content of glucagon in αTC1-6 cells and islets after the expression of control shRNA and shRNA-IRKD. (**A, C**) Glucagon secretion in αTC1-6 cells (**A**) or islets (**C**) was assessed following a 2 h incubation in KRB with 1.5, 5.6 or 30 mM glucose after preincubation for 1 h with 5.6 mM glucose. *n* = 6 (**A**) or 3 (**C**) in each group. (**B, D**) The total protein content and total glucagon content in αTC1-6 cells (**B**) or islets (**D**). *n* = 6 (**B**) or 3 (**D**) in each group. The bars represent the means ± SEM; *P<0.05, **P<0.01, control vs. IRKD.

To assess the effects of IR disruption on the α-cell function, we studied the glucose-regulated glucagon release of IRKD cells. Figure 2A shows a comparison of the glucagon secretion in response to varying glucose concentrations (from 1.5 to 30 mM) between control and IRKD cells. The IRKD cells tended to secrete less glucagon compared to the controls at the 1.5 mM glucose concentration (*P* = 0.13). On the other hand, the IRKD cells exhibited significantly higher glucagon secretion in response to the 5.6 and 30 mM glucose concentrations than controls. The glucagon content in the cells was not significantly different between the control and IRKD cells at any of the three different glucose concentrations (Fig. 2B).

In islets with IRKD, the glucagon secretion in response to 1.5 mM glucose concentration was significantly decreased, while at the 30 mM glucose concentration, the glucagon secretion tended to be increased (*P* = 0.06) compared to the islets expressing control shRNA (Fig. 2C). The glucose-stimulated glucagon content in the islets was not affected by the knockdown of IR expression (Fig. 2D).

### Differences between the metabolic profiles of the control and IRKD αTC1-6 cells detected using CE-TOFMS

**Figure 3:**
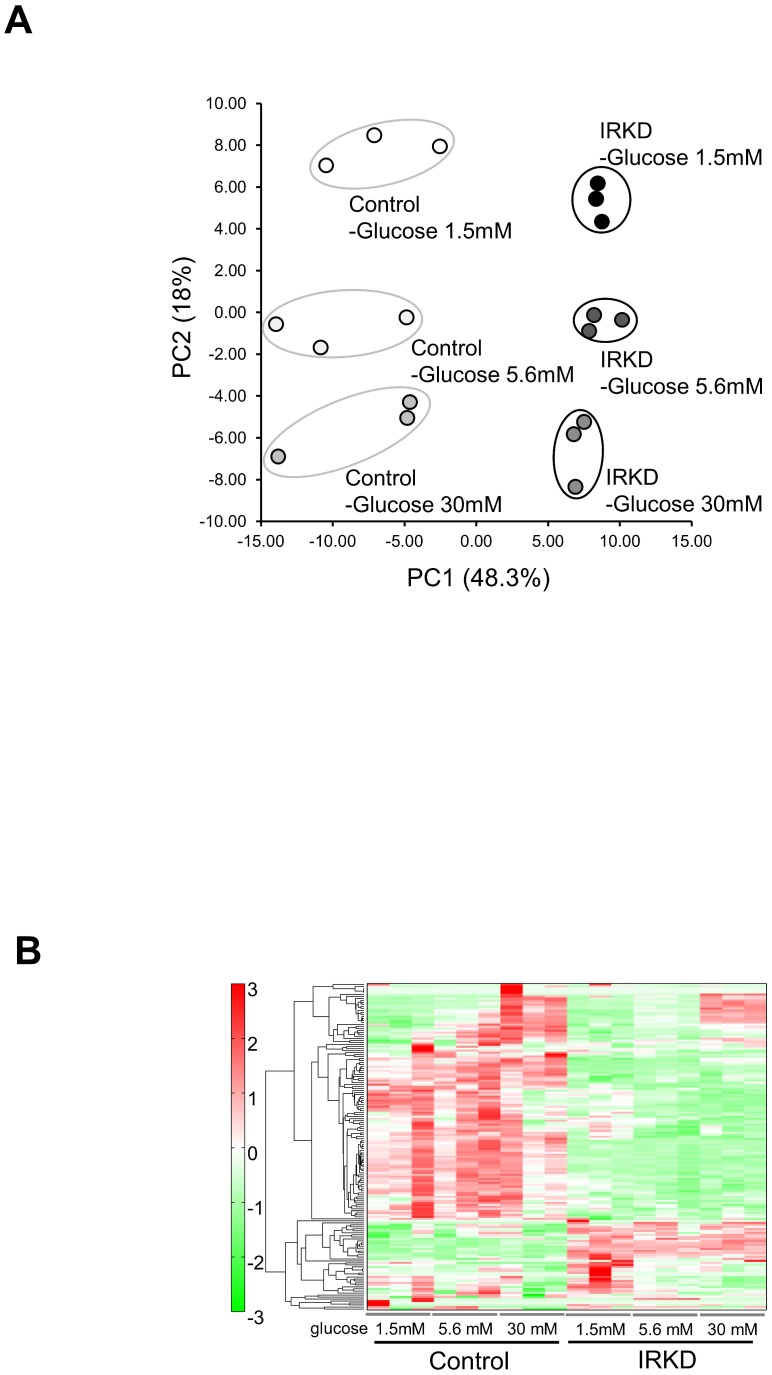
Comparison of the metabolic profile of control and IRKD αTC1-6 cells, as determined by CE-TOFMS. A principal component analysis (PCA) score plot (**A**) and heat map (**B**) from the metabolic data of the control and IRKD αTC1-6 cells treated with three different concentrations of glucose (1.5, 5.6 or 30 mM) (*n* = 3 in each group).

To further investigate the metabolic traits that underlie the abnormal glucagon secretion in IR knockdown α cells, we analyzed the metabolomic profiles of control and IRKD cells using CE-TOFMS. Based on their *m/z* values and migration times, 163 metabolites (75 cations and 88 anions) were detected and visualized on a metabolome-wide pathway map using the VANTED (Visualization and Analysis of Networks containing Experimental Data) software program (http://vanted.ipk-gatersleben.de/). The metabolic pathways associated with all the detected metabolites are illustrated in Figure S1. These metabolites were associated with glycolysis/gluconeogenesis, the pentose phosphate pathway, the TCA cycle, the urea cycle, pyrimidine metabolism, nicotinate and nicotinamide metabolism and amino acid metabolism. The results of the comparison of the metabolic profiles between the control and IRKD cells are provided in Figure 3.

**Figure 4:**
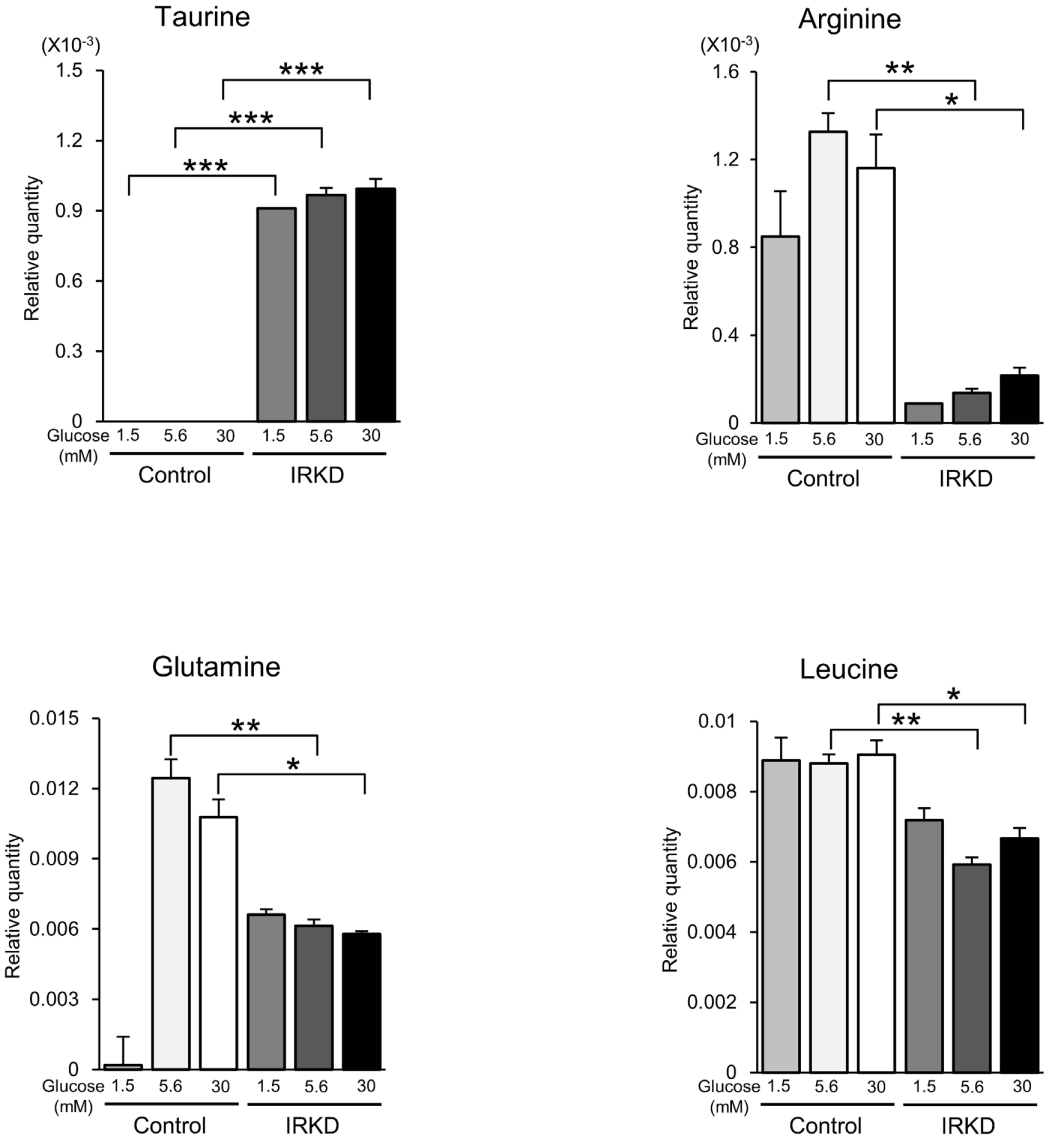
Metabolic quantities of taurine, arginine, glutamine and leucine between control and IRKD αTC1-6 cells. A comparison was made between control and IRKD αTC1-6 cells at three different concentrations of glucose (1.5, 5.6, 30 mM). *n* = 3 in each group. The bars represent the means ± SEM; *P<0.05, **P<0.01, control vs. IRKD.

A principal component analysis (PCA) showed a clear distinction between the intracellular metabolites of control and IRKD cells according to glucose concentrations (Fig. 3A). The first component (PC1) indicated that 48.3% of the total variation was due to the differences between control and IRKD cells. Furthermore, a heatmap analysis indicated that the metabolic pattern of IRKD cells was remarkably different from that of the control cells (Fig. 3B). The cellular contents of 33 metabolites in the control and IRKD cells were significantly different, with P values less than 0.05 for at least two points at each concentration of glucose (Table 1). We observed a decrease of some metabolites associated with the pentose phosphate, TCA cycle, purine-pyrimidine and amino acid metabolism in αTC1-6 cells due to knocking down the IR. In contrast, taurine and choline were relatively increased in the IRKD cells. Of the various metabolites identified as being significantly altered, taurine, arginine, glutamine and leucine (Fig. 4) were chosen for a further analysis because they could potentially affect the glucagon secretion.

**Table 1:** A comparison of the biosynthetic metabolites between the control and IRKD αTC1-6 cells, as determined by CE-TOFMS.

Biosynthetic pathway(s)	Metabolite	Control-1.5	Control-5.6	Control-30	IRKD-1.5	IRKD-5.6	IRKD-30	Control-1.5 vs. IRKD-1.5	Control-5.6 vs. IRKD-5.6	Control-30 vs. IRKD-30
		Mean ± SEM	Mean ± SEM	Mean ± SEM	Mean ± SEM	Mean ± SEM	Mean ± SEM	Ratio¶	Ratio¶	Ratio¶
Glycolysis/gluconeogenesis, pentose phosphate pathway, TCA cycle	Sedoheptulose 7-phosphate	2.3E-04±1.4E-05	2.6E-04±3.5E-05	3.1E-04±8.7E-06	1.4E-04±4.8E-06	1.6E-04±5.1E-06	1.9E-04±8.6E-06	0.6*	0.6	0.62***
	Ribulose 5-phosphate	1.6E-04±1.6E-05	1.7E-04±1.3E-05	2.2E-04±3.0E-05	5.7E-05±3.2E-06	5.9E-05±5.0E-06	1.4E-04±4.0E-06	0.36*	0.36**	0.61
	PRPP	3.4E-04±4.4E-05	4.7E-04±4.7E-05	3.9E-04±1.8E-05	3.2E-05±0.0E+00	5.5E-05±4.5E-06	5.3E-05±8.4E-06	0.09	0.12*	0.13***
	*cis*-Aconitic acid	1.2E-03±1.5E-04	1.8E-03±1.1E-04	1.9E-03±1.1E-04	8.6E-04±1.2E-04	1.1E-03±5.1E-05	1.2E-03±3.6E-05	0.71	0.6*	0.63*
	2-Oxoglutaric acid	1.3E-03±4.1E-05	1.7E-03±1.2E-04	1.7E-03±9.2E-05	8.0E-04±3.3E-05	9.1E-04±4.9E-05	8.6E-04±6.6E-05	0.6***	0.55*	0.52**
Purine metabolism, pyrimidine metabolism	UDP	2.7E-03±4.9E-05	2.1E-03±9.5E-05	1.3E-03±2.0E-04	1.3E-03±8.3E-05	1.0E-03±7.0E-05	7.4E-04±2.0E-05	0.47***	0.5**	0.57
	GMP	3.4E-04±3.9E-06	3.1E-04±2.1E-05	2.6E-04±2.9E-05	2.5E-04±6.0E-06	1.9E-04±1.0E-05	1.9E-04±6.8E-06	0.73***	0.61*	0.73
	GDP	1.5E-03±4.6E-05	1.3E-03±9.4E-05	9.3E-04±1.1E-04	8.5E-04±5.8E-05	8.4E-04±3.6E-05	6.7E-04±3.6E-05	0.58**	0.65*	0.72
	CDP	7.1E-04±1.8E-05	6.1E-04±6.3E-05	4.0E-04±3.9E-05	3.9E-04±3.8E-05	2.8E-04±2.0E-05	2.1E-04±5.9E-06	0.55**	0.46*	0.54*
	UMP	7.0E-04±4.0E-05	6.8E-04±1.0E-04	4.1E-04±9.6E-06	3.2E-04±8.5E-06	2.5E-04±1.5E-05	2.1E-04±1.4E-05	0.45**	0.38	0.52***
	ADP	1.3E-02±3.9E-04	1.2E-02±1.5E-03	8.0E-03±5.7E-04	7.7E-03±7.6E-04	7.1E-03±4.3E-04	5.0E-03±9.7E-05	0.61*	0.58	0.63*
	CTP	8.8E-03±6.1E-04	1.1E-02±1.4E-03	1.0E-02±5.6E-04	5.5E-03±9.4E-05	5.0E-03±1.6E-04	5.1E-03±1.3E-04	0.63*	0.48	0.51**
	CMP	3.0E-04±2.1E-05	2.8E-04±2.3E-05	2.4E-04±1.5E-05	1.9E-04±1.8E-06	1.5E-04±8.1E-06	1.4E-04±8.3E-06	0.61*	0.55*	0.6**
	UTP	2.8E-02±2.1E-03	3.0E-02±3.3E-03	2.6E-02±9.0E-04	1.8E-02±5.2E-04	1.7E-02±6.4E-04	1.7E-02±5.1E-05	0.65*	0.56	0.64**
	dCTP	4.6E-04±2.2E-05	4.5E-04±2.8E-05	4.3E-04±7.0E-06	3.7E-04±1.9E-05	3.4E-04±5.5E-06	3.6E-04±3.6E-06	0.82*	0.75	0.83**
Amino acid metabolism (Asp, Ala, Lys)	Asp	2.2E-01±1.7E-02	1.8E-01±7.5E-03	1.2E-01±1.3E-02	1.4E-01±6.2E-03	1.1E-01±4.1E-04	8.5E-02±1.6E-03	0.65*	0.62*	0.74
	Lys	1.4E-02±1.2E-03	1.3E-02±3.0E-04	1.2E-02±9.1E-04	9.3E-03±2.6E-04	7.9E-03±3.8E-04	7.5E-03±2.9E-04	0.69	0.6***	0.64*
	*O*-Acetylcarnitine	5.2E-04±7.8E-05	9.9E-04±5.1E-05	1.3E-03±7.1E-05	6.0E-04±3.8E-05	7.5E-04±4.1E-05	9.0E-04±4.3E-05	1.17	0.76*	0.67*
Amino acid metabolism (Gly, Ser, Cys)	CDP-choline	1.3E-04±7.4E-06	1.7E-04±2.3E-05	1.6E-04±2.0E-05	3.6E-04±5.5E-06	3.4E-04±1.8E-05	4.0E-04±2.9E-05	2.73***	2.05**	2.46**
	Choline	1.2E-02±5.7E-04	1.3E-02±2.2E-04	1.5E-02±5.6E-04	2.7E-02±2.4E-03	2.3E-02±1.2E-03	2.9E-02±1.7E-03	2.29*	1.82*	1.98**
	Hypotaurine	3.8E-03±4.6E-04	4.0E-03±2.4E-04	3.6E-03±2.7E-04	6.8E-03±3.5E-04	6.6E-03±4.3E-04	6.1E-03±1.2E-04	1.77**	1.66*	1.69**
	Taurine	0.0E+00±0.0E+00	0.0E+00±0.0E+00	0.0E+00±0.0E+00	9.1E-04±0.0E+00	9.7E-04±3.0E-05	9.9E-04±4.1E-05	1<***	1<***	1<***
Amino acid metabolism (Branched chain)	Val	9.9E-03±7.2E-04	1.0E-02±5.0E-04	9.9E-03±3.5E-04	7.8E-03±2.1E-04	7.0E-03±2.2E-04	7.7E-03±1.5E-04	0.79	0.69*	0.78*
	Leu	8.9E-03±6.5E-04	8.8E-03±2.6E-04	9.1E-03±4.1E-04	7.2E-03±3.4E-04	5.9E-03±2.1E-04	6.7E-03±3.0E-04	0.81	0.67**	0.74*
	Ile	4.0E-03±3.1E-04	4.0E-03±2.1E-04	4.1E-03±9.3E-05	3.4E-03±2.1E-04	2.6E-03±9.5E-05	3.3E-03±8.3E-05	0.86	0.66*	0.81**
Urea cycle, amino acid metabolism (Glu, Gln, His, Pro)	Argininosuccinic acid	4.8E-04±5.5E-05	5.2E-04±3.3E-05	4.5E-04±6.0E-05	2.1E-04±1.3E-05	2.0E-04±1.3E-05	2.1E-04±1.5E-05	0.42*	0.38**	0.46*
	Arg	8.5E-04±2.1E-04	1.3E-03±8.6E-05	1.2E-03±1.5E-04	8.8E-05±0.0E+00	1.4E-04±1.9E-05	2.2E-04±3.6E-05	0.1	0.1**	0.19*
	Guanidoacetic acid	8.2E-04±7.5E-05	8.6E-04±4.3E-05	8.1E-04±4.5E-05	4.6E-04±1.7E-05	4.1E-04±3.0E-05	3.7E-04±3.2E-05	0.56*	0.48**	0.46**
	Pro	1.0E-01±7.9E-03	1.0E-01±6.8E-03	9.1E-02±6.4E-03	6.6E-02±1.8E-03	5.8E-02±2.6E-03	5.8E-02±2.6E-03	0.66*	0.57*	0.64*
	Gln	1.2E-02±1.2E-03	1.2E-02±8.1E-04	1.1E-02±7.6E-04	6.6E-03±2.4E-04	6.1E-03±2.7E-04	5.8E-03±1.1E-04	0.57	0.49**	0.54*
	5-Oxoproline	3.4E-04±3.6E-05	3.4E-04±1.9E-05	3.8E-04±3.1E-05	2.5E-04±1.7E-05	2.1E-04±4.2E-06	1.8E-04±5.5E-06	0.73	0.6*	0.46*
	Ornithine	4.2E-03±3.8E-04	4.3E-03±1.7E-04	4.1E-03±2.4E-04	3.4E-03±7.9E-05	2.8E-03±1.6E-04	2.7E-03±1.8E-04	0.81	0.64**	0.66*
	His	2.7E-03±3.3E-04	2.8E-03±4.8E-05	2.7E-03±2.5E-04	2.1E-03±1.3E-04	1.8E-03±9.5E-05	1.8E-03±1.3E-04	0.77	0.66**	0.65*

Control, shRNA control αTC1-6 cells; IRKD, shRNA insulin receptor knockdown αTC1-6 cells; −1.5, treated with 1.5 mM glucose; −5.6, treated with 5.6 mM glucose; −30, treated with 30 mM glucose.

*P<0.05,

**P<0.01,

***P<0.001 (Control vs. IRKD).

¶ Control/IRKD ratio.

### The effects of IR knockdown on the taurine and amino acid uptake and synthesis

**Figure 5:**
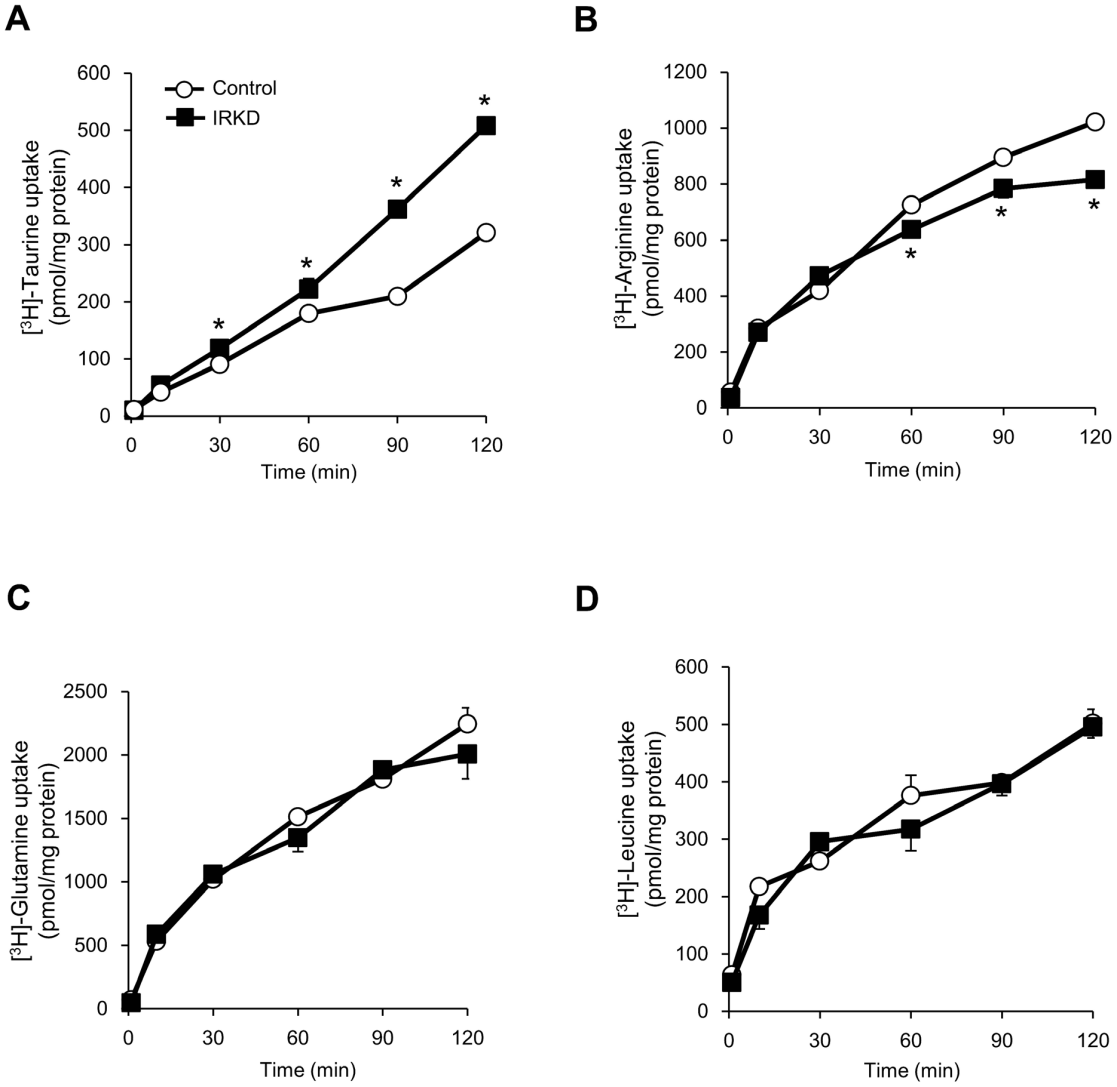
Time course of [^3^H] taurine and amino acid uptake in control and IRKD αTC1-6 cells. *n* = 4 in each group. The data are expressed as the means ± SEM; *P<0.05, control vs. IRKD.

We examined whether IR knockdown affected the uptake and synthesis of taurine, arginine, glutamine and leucine in αTC1-6 cells. Figure 5A shows the time-dependent [^3^H] taurine uptake for 120 min in the control and IRKD cells. The IRKD cells exhibited significantly higher levels of taurine uptake than control cells from 30 to 120 min. On the other hand, the arginine uptake in the IRKD cells was shown to decrease from 60 to 120 min compared with the controls (Fig. 5B). There were no significant differences in the glutamine and leucine uptake between the IRKD and control cells (Figs. 5C and D). It is noteworthy that the uptake of labeled nutrients that are metabolized into CO_2_, H_2_O and other secreted metabolites would not be included in the measurement, because the assay measures the radioactivity trapped in the cells, so the values obtained in this study may have underestimated the actual values.

**Figure 6:**
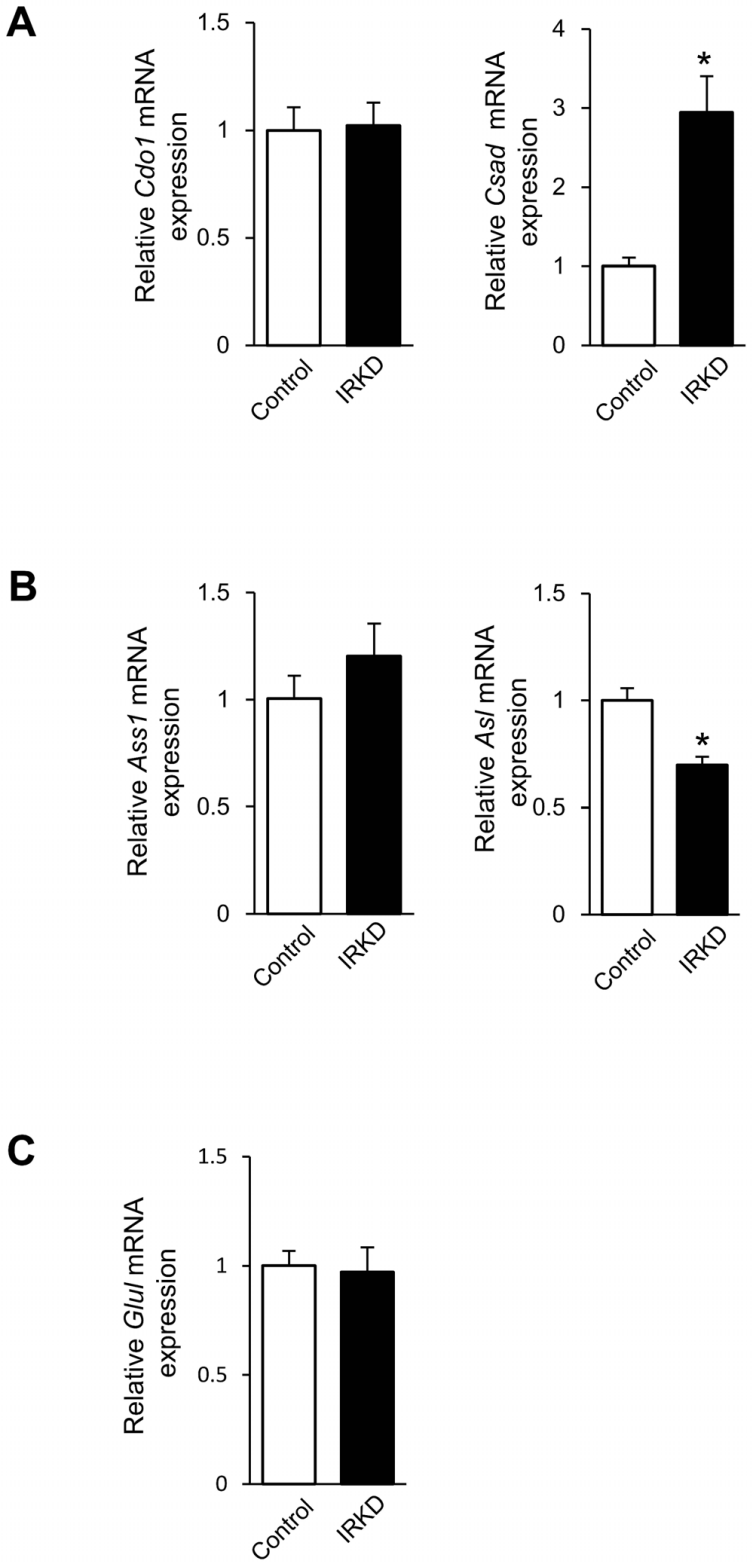
Altered gene expression of the major enzymes required for taurine, arginine and glutamine synthesis in IRKD αTC1-6 cells. The relative expression of *Cdo1*, *Csad*, *Ass1*, *Asl* and *Glul* genes in the control and IRKD αTC1-6 cells incubated for 3 h with KRB containing 5.6 mM glucose was measured by quantitative real-time PCR and normalized to the expression of the reference gene, *Actb*. *n* = 4 in each group. The bars represent the means ± SEM; *P<0.05, control vs. IRKD.

Next, we compared the enzymes involved in taurine, arginine and glutamine metabolism in control and IRKD cells (Fig. 6). The gene expression levels of the major synthetic enzymes for taurine (cysteine dioxygenase 1 (*Cdo1*), which converts cysteine to cysteine sulfinic acid, and cysteine sulfinic acid decarboxylase (*Csad*), which converts cysteine sulfinic acid to hypotaurine); arginine (arginosuccinate synthase 1 (*Ass1*), which converts citrulline to argininosuccinate, and argininosuccinate lyase (*Asl*), which converts argininosuccinate to arginine) and glutamine (glutamine synthase (*Glul*)) were examined. As shown in Fig. 6A, the expression of *Csad*, the rate-limiting enzyme required for taurine synthesis, was significantly elevated in IRKD cells compared with the control cells. In contrast, the mRNA expression of *Asl*, an enzyme that catalyzes the final step of arginine biosynthesis, was decreased in IRKD cells (Fig. 6B). We found no significant difference in the *Glul* expression between IRKD and control cells (Fig. 6C).

### Effects of taurine on the glucagon secretion by αTC1-6 cells and islets

**Figure 7:**
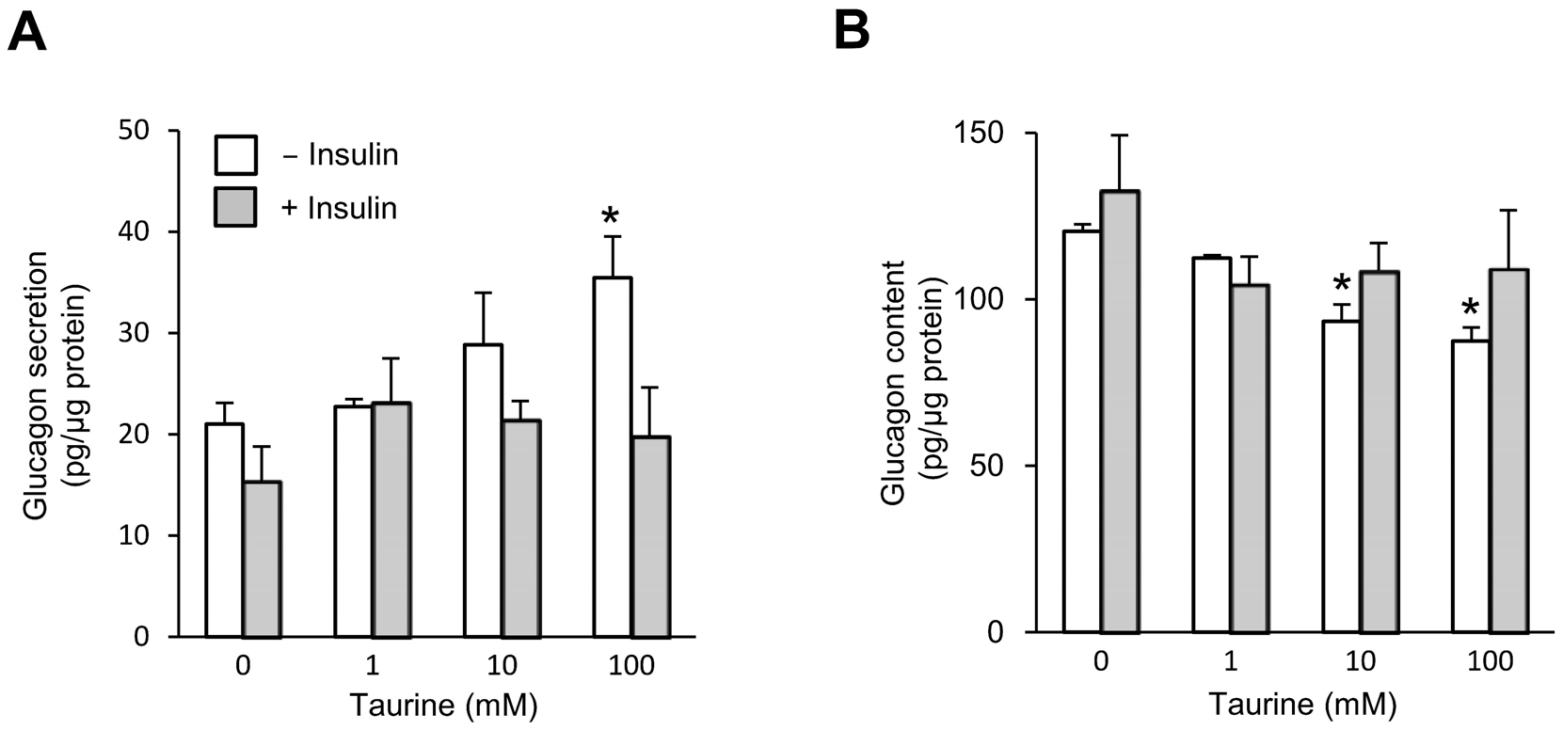
Taurine-stimulated glucagon secretion and cell contents in control αTC1-6 cells with or without insulin. (**A**) Cells were preincubated for 1 h with KRB containing 5.6 mM glucose, and were subsequently stimulated for 2 h with 0, 1, 10 or 100 mM taurine and treated with or without insulin (100 nM). *n* = 6 in each group. (**B**) The total protein content and total glucagon content. *n* = 6 in each group. The bars represent the means ± SEM; *P<0.05, vs. vehicle-treated cells.

Because taurine was considered to be one of the most likely candidate metabolite stimulating glucagon secretion, we determined the effects of taurine supplementation on the glucagon release and synthesis. Consequently, supplementation of taurine enhanced the glucagon secretion in a concentration-dependent manner in the αTC1-6 cells, and this increase in glucagon secretion by taurine was inhibited by adding insulin (Fig. 7A). On the other hand, taurine concentration-dependently reduced the glucagon content in these cells, and the reduction disappeared with the addition of insulin (Fig. 7B). This result suggests that insulin suppresses the stimulatory effect of taurine on glucagon secretion in the αTC1-6 cells.

**Figure 8:**
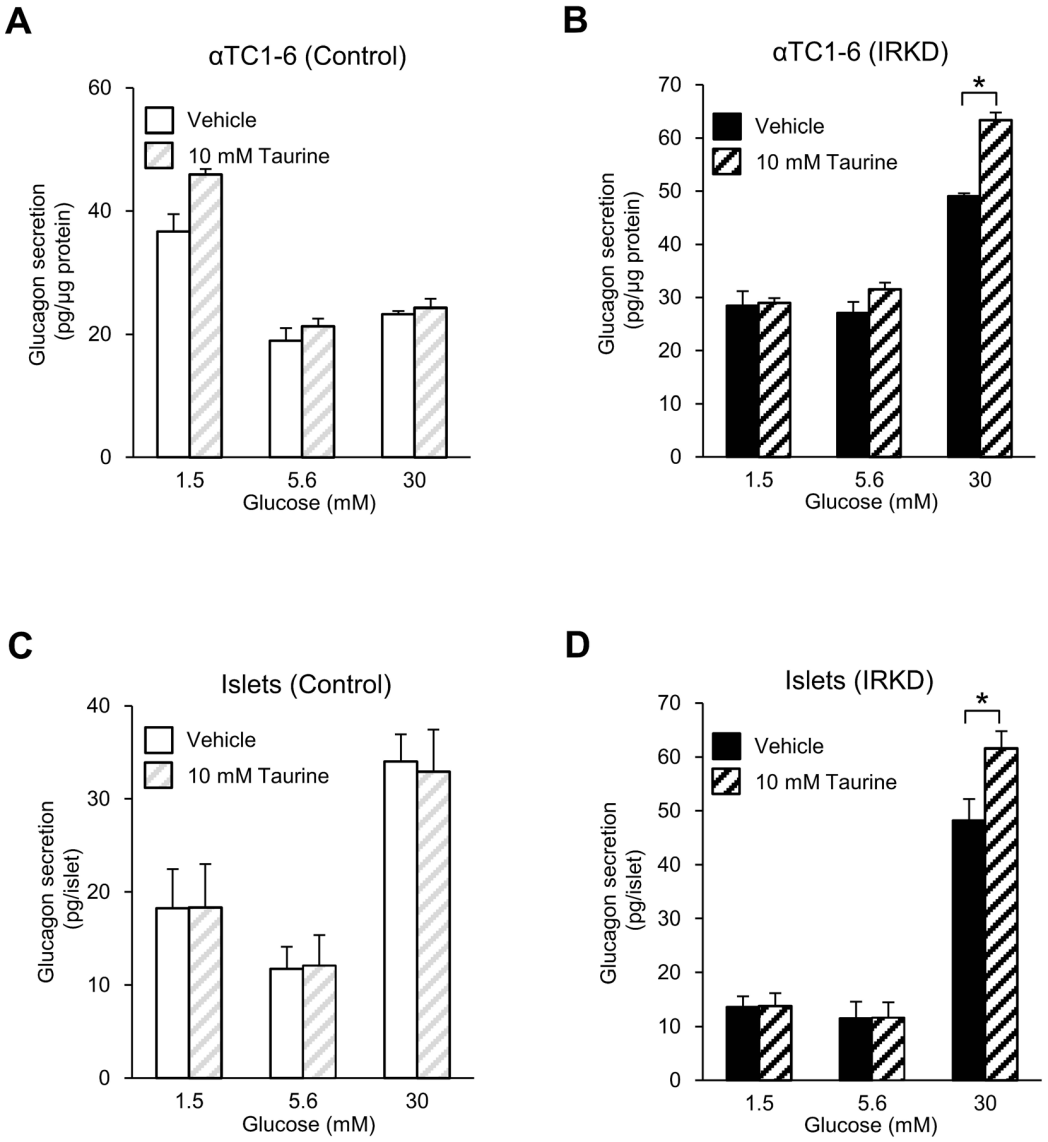
Exaggerated glucose-stimulated glucagon secretion in IRKD αTC1-6 cells and islets in the presence of taurine. αTC1-6 cells (**A, B**) or islets (**C, D**) were preincubated for 1 h with KRB containing 5.6 mM glucose, and were subsequently stimulated for 2 h with 1.5, 5.6 or 30 mM glucose with or without 10 mM taurine. *n* = 6 (**A, B**) or 3 (**C, D**) in each group. The bars represent the means ± SEM; *P<0.05, taurine-treated vs. vehicle-treated cells or islets.

We also assessed the glucagon secretion in response to three different concentrations of glucose (1.5, 5.6 and 30 mM) with or without 10 mM taurine in control and IRKD cells. Since 10 mM taurine supplementation tended to increase the glucagon release in control αTC1-6 cells (albeit not reaching statistical significance at the 5.6 mM glucose level), and thus could be assumed to enhance glucagon secretion depending upon the glucose concentration, the treatment with a 10 mM concentration of taurine was subsequently examined in IRKD cells and islets. The treatment with 10 mM taurine had no effect on the glucagon released by the control αTC1-6 cells and control islets (Figs. 8A and C); however, when both αTC1-6 cells and islets with knocked down IR were treated with 30 mM glucose plus 10 mM taurine, the glucagon secretion was significantly augmented compared with that in vehicle-treated ones (Figs. 8B and D).

## Discussion

This study revealed that there were differential levels of metabolites, together with the abnormal glucagon response to glucose, in an *in vitro* model of intra-islet insulin-deficient α-cells. Notably, taurine was found to be markedly elevated, and was shown to stimulate glucagon secretion in the α-cells, thus suggesting that it might exaggerate the paradoxical secretion of glucagon from α-cells under conditions of insulin deficiency.

We succeeded in generating an αTC1-6 cell line with stable knockdown of the IR. αTC1-6 is a clonal murine α-cell line that specifically produces glucagon but not insulin [17]. This cell line possesses an advantage over primary islets in that it is a homogeneous cellular population and has been used previously to study glucagon secretion and gene expression [15], [17]–[19], [27]. IR knockdown in stable α-cell lines could reproduce the circumstances reflecting a chronic deficiency of extracellular insulin, which may reflect the pathophysiology of α-cells in insulin deficiency. Namely, in insulin deficiency, where β-cells are lost, α-cells lack the tonic restraint normally provided by the high concentrations of insulin from juxtaposed β-cells [3]. Clinical studies have shown that in type 1 diabetes, glucose, administered either intravenously or orally, can actually stimulate glucagon secretion [28], while the glucagon response to low glucose is blunted [29], [30], indicating that these “opposite” or “paradoxical” changes in the glucagon dynamics may lead to disrupted glucose homeostasis. Thus, exploring the mechanisms responsible for glucose-regulated glucagon dysfunction in an insulin-deficient α-cell model may shed a light on the pathogenesis of glycemic instability in insulin-deficient type 1 diabetes.

In this study, IRKD cells exhibited paradoxical glucagon behavior at different concentrations of glucose. The islets with IRKD also showed a paradoxical pattern, although the statistically different points found in islets and αTC1-6 cells were different; the glucagon secretion in response to low glucose was significantly abolished in islets with IRKD, while that in response to high glucose was significantly exaggerated in the αTC1-6 cells with IRKD. This might have been due to the phenotypic differences between the isolated islets and αTC1-6 cells, or to the differences caused by the transient or stable knock-down of the IR. Diao et al. [15] have previously reported that glucagon secretion is abolished in response to low glucose in isolated islets and in αTC1-6 cells expressing insulin receptor siRNA, whereas glucagon release at the high glucose concentration remained the same as in the control cells. The difference between these two studies may be explained by the different concentrations of glucose used during the preincubation period (5.6 mM in our study and 25 mM in the latter study). Another previous study showed that there was paradoxical glucagon gene expression under both high- and low-glucose conditions in the islets of α-cell specific insulin receptor knockout mice [16]. Furthermore, siRNA-mediated knockdown of the IR in glucagon-secreting InR1G cells promoted enhanced glucagon secretion [16]. Our data are consistent with these findings, and may support the idea that IR expression is essential for glucose-regulated glucagon release.

A novel finding of the present study is the distinct metabolomic patterns between the IRKD and control αTC1-6 cells. The TCA cycle metabolites, such as *cis*-aconitic acid and 2-oxoglutaric acid, the metabolites of the pentose phosphate and nucleotide synthesis pathways and several essential and conditionally essential amino acids were decreased in IRKD cells. These results may reflect the reduced bioenergetic and biosynthetic requirements in IRKD cells due to their decreased viability and proliferation. Regarding the effect of the glucose concentration, no meaningful metabolic differences were seen between IRKD and control cells. The most remarkable difference between the two types of cells was that taurine was abundant in IRKD cells, while it was almost undetectable in control αTC1-6 cells. The quantities of taurine at different glucose concentrations were similar, although the effects of taurine on glucagon secretion were different at the different glucose concentrations in the IRKD cells. This may suggest that intracellular and extracellular taurine have different effects on glucagon secretion at the same glucose concentrations.

Of the various metabolites evaluated, taurine, arginine, glutamine and leucine were considered to potentially exert some effects on the glucagon secretion. Taurine has been reported to inactivate the ATP-sensitive K^+^-channels either directly via an interaction with the sulfonylurea receptor (SUR) on the channel, or indirectly via an associated protein that binds to the SUR in skeletal muscle fibers [31]. In neurons and cardiac cells, taurine-modulated calcium flux has been reported [32], [33]. Furthermore, it has been proposed that the administration of taurine leads to the opening of voltage-gated calcium channels and a subsequent decrease in the intracellular insulin levels in β-cell lines [34]. Together, these data suggest that taurine enhances glucagon release partly by altering the electrogenic response in α-cells. Our data showing an increase of taurine uptake and synthesis in IRKD cells compared with control cells are consistent with the metabolomic results of the comparatively elevated cellular taurine level in IRKDs, and may account for their exaggerated glucagon response under the high glucose condition.

The intracellular amino acids, arginine, glutamine and leucine, were assumed to play a role in glucagon release, at least in part, through the activation or inactivation of AMPK and mTOR, which are two important energy/nutrient centers [35]. mTOR has been proposed as a downstream component of an energy-sensing pathway, with AMPK as the primary cellular energy sensor [36]. Increases in AMPK activity stimulate glucagon secretion in α-cells [37]. However, both the AMPK and mTOR activity were unchanged after the removal of IR in the present study, which is in accordance with the results of a previous study [15]. Cellular arginine is known to induce glucagon release through a mechanism mediated by nitric oxide [38], [39], regardless of the glucose concentration [40]. Thus, the decreased arginine uptake and synthesis observed in IRKD cells may partly contribute to the inhibition of glucagon release at the low glucose concentration in these cells.

Finally, taurine was shown to stimulate glucagon secretion in αTC1-6 cells. Taurine is present in most mammalian tissues, including the pancreatic islets, and regulates the osmolarity, ion channel activity and glucose homeostasis. It has been shown that taurine administration strongly suppresses the glucose-stimulated secretion of insulin from isolated mouse islets [41], and also that the intraperitoneal injection of taurine inhibits the increase in serum insulin induced by glucose administration [42]. On the contrary, it has been reported that taurine enhances glucose-stimulated insulin release from cultured rat fetal islets [43]. Another report showed that taurine supplementation to mice improved the insulin secretion and enhanced glucagon secretion from isolated islets [44]. The results showing that the extracellular taurine concentration-dependently reduced the glucagon content in αTC1-6 cells may reflect a relative excess of glucagon exocytosis compared to its biosynthesis. Together, these data indicate a possible glucagonotropic action of taurine in α-cells.

Our results also showed that taurine paradoxically exaggerated the glucagon secretion under the high glucose concentration in both IRKD cells and islets with IRKD, suggesting that it could worsen the aberrant α-cell function under the condition of chronic insulin deficiency. Taurine is known to move passively through the anionic volume regulatory channels [45]. At the 30 mM glucose concentration, the uptake of supplemented taurine into IRKD cells would be higher, because of its high osmolarity, in order to regulate the cell volume compared to that at the 1.5 or 5.6 mM glucose concentration. Therefore, the effects of taurine supplementation on glucagon secretion may be elicited only at 30 mM glucose in IRKD cells and islets. Glucose elevation reduces the activation of both K_ATP_ channels and the P/Q-type Ca^2+^-current, which accordingly decrease the glucagon secretion at high glucose levels in physiological α-cells [46]. When insulin signaling is impaired, the K_ATP_ and Ca^2+^ channel activity or sensitivity is altered [47]. Taurine might therefore play a role in the glucagon secretion from IRKD cells by affecting the K_ATP_ and Ca^2+^ channels. Conceivably, the combination of a high glucose (30 mM) concentration and 10 mM taurine supplementation can “optimize” the membranous electrical activity for glucagon exocytosis in IRKD cells and islets. However, all of these causal mechanisms are only speculative, and therefore, should be fully evaluated in the future.

It is notable that taurine was accumulated, and may contribute to paradoxical glucagon secretion in this insulin-deficient α-cell model. While some beneficial effects of taurine supplementation for reducing blood glucose and restoring insulin sensitivity have been found in type 2 diabetes, in type 1 diabetes, taurine might exert adverse effects on the glucose stability as a result of α-cell dysfunction.

In conclusion, our study indicates that the metabolic alterations induced by IRKD in α-cells, especially the increase of taurine, may lead to a distorted glucagon response in IRKD αTC1-6 cells, i.e., an *in vitro* model of insulin-deficient α-cells, thus suggesting the importance of taurine in the paradoxical glucagon response and the resultant glucose instability in insulin-deficient type 1 diabetes. Further *in vivo* studies to assess the long-term relevance of taurine to the glucagon secretion and glycemic control in insulin-deficient type 1 diabetes are needed to investigate the clinical implications of our findings.

## Supporting Information

Figure S1 Metabolites in the principle metabolic pathways of control and IRKD αTC1-6 cells detected by CE-TOFMS.
(TIF)

## References

[1] Reynolds C, Molnar GD, Horwitz DL, Rubenstein AH, Taylor WF, et al (1997) Abnormalities of endogenous glucagon and insulin in unstable diabetes. Diabetes 26: 36–45. 10.2337/diab.26.1.36 830563

[2] The Diabetes Control and Complications Trial Research Group (1998) Effect of intensive therapy on residual beta-cell function in patients with type 1 diabetes in the diabetes control and complications trial. A randomized, controlled trial. Ann Intern Med 128: 517–523. 9518395 10.7326/0003-4819-128-7-199804010-00001

[3] Unger RH, Orci L (2010) Paracrinology of islets and the paracrinopathy of diabetes. Proc Natl Acad Sci U S A 107: 16009–16012. 20798346 10.1073/pnas.1006639107 PMC2941311

[4] Siafarikas A, Johnston RJ, Bulsara MK, O'Leary P, Jones TW, et al (2012) Early loss of the glucagon response to hypoglycemia in adolescents with type 1 diabetes. Diabetes Care 35: 1757–1762. 22699295 10.2337/dc11-2010 PMC3402257

[5] Pörksen S, Nielsen LB, Kaas A, Kocova M, Chiarelli F, et al (2007) Meal-stimulatd glucagon release is associated with postprandial blood glucose level and does not interfere with glycemic control in children and adolescents with new-onset type 1 diabetes. J Clin Endocrinol Metab 92: 2910–2916. 17519307 10.1210/jc.2007-0244

[6] Bessho M, Murase-Mishiba Y, Tsutsumi C, Haseda F, Imagawa A, et al (2013) Glycaemic instability correlates with a hyperglucagonaemic response in patients with type 1 diabetes without residual beta-cell function. Diabetes Res Clin Pract 102: e38–e40. 24095157 10.1016/j.diabres.2013.09.003

[7] Taborsky GJ Jr (2010) The physiology of glucagon. J Diabetes Sci Technol 4: 1338–1344. 21129328 10.1177/193229681000400607 PMC3005043

[8] Gaisano HY, Macdonald PE, Vranic M (2012) Glucagon secretion and signaling in the development of diabetes. Front Physiol 3: e349. 10.3389/fphys.2012.00349 PMC3432929 22969729

[9] Gerich JE, Tsalikian E, Lorenzi M, Schneider V, Bohannon NV, et al (1975) Normalization of fasting hyperglucagonemia and excessive glucagon responses to intravenous arginine in human diabetes mellitus by prolonged infusion of insulin. J Clin Endocrinol Metab 41: 1178–1180. 1206104 10.1210/jcem-41-6-1178

[10] Wendt A, Birnir B, Buschard K, Gromada J, Salehi A, et al (2004) Glucose inhibition of glucagon secretion from rat alpha-cells is mediated by GABA released from neighboring beta-cells. Diabetes 53: 1038–1045. 15047619 10.2337/diabetes.53.4.1038

[11] Rorsman P, Berggren PO, Bokvist K, Ericson H, Möhler H, et al (1989) Glucose-inhibition of glucagon secretion involves activation of GABAA-receptor chloride channels. Nature 341: 233–236. 2550826 10.1038/341233a0

[12] Ishihara H, Maechler P, Gjinovci A, Herrera PL, Wollheim CB (2003) Islet beta-cell secretion determines glucagon release from neighbouring alpha-cells. Nat Cell Biol 5: 330–335. 12640462 10.1038/ncb951

[13] Kawamori D, Akiyama M, Hu J, Hambro B, Kulkarni RN (2011) Growth factor signaling in the regulation of α-cell fate. Diabetes Obes Metab 13, Suppl 1: 21–30. 21824253 10.1111/j.1463-1326.2011.01442.x

[14] Bollyky J, Greenbaum CJ (2007) Editorial: The role of glucagon in postprandial hyperglycemia–the jury's still out. J Clin Endocrinol Metab 92: 2879–2881. 17682088 10.1210/jc.2007-1312

[15] Diao J, Asghar Z, Chan CB, Wheeler MB (2005) Glucose-regulated glucagon secretion requires insulin receptor expression in pancreatic alpha-cells. J Biol Chem 280: 33487–33496. 16027126 10.1074/jbc.M506276200

[16] Kawamori D, Kurpad AJ, Hu J, Liew CW, Shih JL, et al (2009) Insulin signaling in alpha cells modulates glucagon secretion in vivo. Cell Metab 9: 350–361. 19356716 10.1016/j.cmet.2009.02.007 PMC2694613

[17] Hamaguchi K, Leiter EH (1990) Comparison of cytokine effects on mouse pancreatic alpha-cell and beta-cell lines. Viability, secretory function, and MHC antigen expression. Diabetes 39: 415–425. 2108069 10.2337/diab.39.4.415

[18] Hayashi M, Otsuka M, Morimoto R, Hirota S, Yatsushiro S, et al (2001) Differentiation-associated Na+-dependent inorganic phosphate cotransporter (DNPI) is a vesicular glutamate transporter in endocrine glutamatergic systems. J Biol Chem 276: 43400–43406. 11551935 10.1074/jbc.M106244200

[19] Hayashi M, Yamada H, Uehara S, Morimoto R, Muroyama A, et al (2003) Secretory granule-mediated co-secretion of L-glutamate and glucagon triggers glutamatergic signal transmission in islets of Langerhans. J Biol Chem 278: 1966–1974. 12414805 10.1074/jbc.M206758200

[20] Yaekura K, Yada T (1998) [Ca2+]i-reducing action of cAMP in rat pancreatic beta-cells: involvement of thapsigargin-sensitive stores. Am J Physiol 274: C513–C521. 9486142 10.1152/ajpcell.1998.274.2.C513

[21] Lambert IH (2004) Regulation of the cellular content of the organic osmolyte taurine in mammalian cells. Neurochem Res 29: 27–63. 14992263 10.1023/b:nere.0000010433.08577.96

[22] Timbrell JA, Seabra V, Waterfield CJ (1995) The in vivo and in vitro protective properties of taurine. Gen Pharmacol 26: 453–462. 7789717 10.1016/0306-3623(94)00203-y

[23] Soga T, Heiger DN (2000) Amino acid analysis by capillary electrophoresis electrospray ionization mass spectrometry. Anal Chem 72: 1236–1241. 10740865 10.1021/ac990976y

[24] Soga T, Ueno Y, Naraoka H, Ohashi Y, Tomita M, et al (2002) Simultaneous determination of anionic intermediates for Bacillus subtilis metabolic pathways by capillary electrophoresis electrospray ionization mass spectrometry. Anal Chem 74: 2233–2239. 12038746 10.1021/ac020064n

[25] Soga T, Ohashi Y, Ueno Y, Naraoka H, Tomita M, et al (2003) Quantitative metabolome analysis using capillary electrophoresis mass spectrometry. J Proteome Res 2: 488–494. 14582645 10.1021/pr034020m

[26] Chang RC, Stadlin A, Tsang D (2001) Effects of tumor necrosis factor alpha on taurine uptake in cultured rat astrocytes. Neurochem Int 38: 249–254. 11099784 10.1016/s0197-0186(00)00082-6

[27] Paul GL, Waegner A, Gaskins HR, Shay NF (1998) Histidine availability alters glucagon gene expression in murine alphaTC6 cells. J Nutr 128: 973–976. 9614156 10.1093/jn/128.6.973

[28] Greenbaum CJ, Prigeon RL, D'Alessio DA (2002) Impaired beta-cell function, incretin effect, and glucagon suppression in patients with type 1 diabetes who have normal fasting glucose. Diabetes 51: 951–957. 11916912 10.2337/diabetes.51.4.951

[29] Gerich JE, Langlois M, Noacco C, Karam JH, Forsham PH (1973) Lack of glucagon response to hypoglycemia in diabetes: evidence for an intrinsic pancreatic alpha cell defect. Science 182: 171–173. 4581053 10.1126/science.182.4108.171

[30] Bergenstal RM, Polonsky KS, Pons G, Jaspan JB, Rubenstein AH (1983) Lack of glucagon response to hypoglycemia in type I diabetics after long-term optimal therapy with a continuous subcutaneous insulin infusion pump. Diabetes 32: 398–402. 6840400 10.2337/diab.32.5.398

[31] Tricarico D, Barbieri M, Camerino DC (2000) Taurine blocks ATP-sensitive potassium channels of rat skeletal muscle fibres interfering with the sulphonylurea receptor. Br J Pharmacol 130: 827–834. 10864889 10.1038/sj.bjp.0703385 PMC1572140

[32] El Idrissi A (2008) Taurine increases mitochondrial buffering of calcium: role in neuroprotection. Amino Acids 34: 321–328. 16955229 10.1007/s00726-006-0396-9

[33] Satoh H, Sperelakis N (1998) Review of some actions of taurine on ion channels of cardiac muscle cells and others. Gen Pharmacol 30: 451–463. 9522160 10.1016/s0306-3623(97)00309-1

[34] L'Amoreaux WJ, Cuttitta C, Santora A, Blaize JF, Tachjadi J, et al (2010) Taurine regulates insulin release from pancreatic beta cell lines. J Biomed Sci 17, Suppl 1: S1–S11. 20804585 10.1186/1423-0127-17-S1-S11 PMC2994409

[35] Newsholme P, Bender K, Kiely A, Brennan L (2007) Amino acid metabolism, insulin secretion and diabetes. Biochem Soc Trans 35: 1180–1186. 17956307 10.1042/BST0351180

[36] Gleason CE, Lu D, Witters LA, Newgard CB, Birnbaum MJ (2007) The role of AMPK and mTOR in nutrient sensing in pancreatic beta-cells. J Biol Chem 282: 10341–10351. 17287212 10.1074/jbc.M610631200

[37] Leclerc I, Sun G, Morris C, Fernandez-Millan E, Nyirenda M, et al (2011) AMP-activated protein kinase regulates glucagon secretion from mouse pancreatic alpha cells. Diabetologia 54: 125–134. 20938634 10.1007/s00125-010-1929-z PMC6101198

[38] Henningsson R, Lundquist I (1998) Arginine-induced insulin release is decreased and glucagon increased in parallel with islet NO production. Am J Physiol 275: E500–E506. 9725818 10.1152/ajpendo.1998.275.3.E500

[39] Henningsson R, Alm P, Lindström E, Lundquist I (2000) Chronic blockade of NO synthase paradoxically increases islet NO production and modulates islet hormone release. Am J Physiol Endocrinol Metab 279: E95–E107. 10893328 10.1152/ajpendo.2000.279.1.E95

[40] Salehi A, Meidute Abaraviciene S, Jimenez-Feltstrom J, Ostenson CG, Efendic S, et al (2008) Excessive islet NO generation in type 2 diabetic GK rats coincides with abnormal hormone secretion and is counteracted by GLP-1. PLoS One 3: e2165. 18478125 10.1371/journal.pone.0002165 PMC2367446

[41] Tokunaga H, Yoneda Y, Kuriyama K (1983) Streptozotocin-induced elevation of pancreatic taurine content and suppressive effect of taurine on insulin secretion. Eur J Pharmacol 87: 237–243. 6220915 10.1016/0014-2999(83)90333-3

[42] Kulakowski EC, Maturo J (1984) Hypoglycemic properties of taurine: not mediated by enhanced insulin release. Biochem Pharmacol 33: 2835–2838. 6383406 10.1016/0006-2952(84)90204-1

[43] Cherif H, Reusens B, Dahri S, Remacle C, Hoet JJ (1996) Stimulatory effects of taurine on insulin secretion by fetal rat islets cultured in vitro. J Endocrinol 151: 501–506. 8994395 10.1677/joe.0.1510501

[44] Ribeiro RA, Bonfleur ML, Amaral AG, Vanzela EC, Rocco SA (2009) Taurine supplementation enhances nutrient-induced insulin secretion in pancreatic mice islets. Diabetes Metab Res Rev 25: 370–379. 19405082 10.1002/dmrr.959

[45] Brès V, Hurbin A, Duvoid A, Orcel H, Moos FC, et al (2000) Pharmacological characterization of volume-sensitive, taurine permeable anion channels in rat supraoptic glial cells. Br J Pharmacol 130: 1976–1982. 10952690 10.1038/sj.bjp.0703492 PMC1572259

[46] Zhang Q, Ramracheya R, Lahmann C, Tarasov A, Bengtsson M, et al (2013) Role of KATP channels in glucose-regulated glucagon secretion and impaired counterregulation in type 2 diabetes. Cell Metab 18: 871–882. 24315372 10.1016/j.cmet.2013.10.014 PMC3851686

[47] Leung YM, Ahmed I, Sheu L, Gao X, Hara M, et al (2006) Insulin regulates islet alpha-cell function by reducing K_ATP_ channel sensitivity to adenosine 5′-triphosphate inhibition. Endocrinology 147: 2155–2162. 16455778 10.1210/en.2005-1249

